# Supplementary material for: A UMLS-based spell checker for natural language processing in vaccine safety
Source: BMC Med Inform Decis Mak. 2007 Feb 12;7:3. doi: 10.1186/1472-6947-7-3 (PMC1805499; doi:10.1186/1472-6947-7-3)
Supplement: Additional file 13 — RAP application directory. Sets up directory for RAP (RDF application for PHP) source code [file 1472-6947-7-3-S13.gz › rap/doc/phpdoc/__filesource/fsource_ontModel__ontModelOWLVocabulary.php.html]

xml version="1.0" encoding="iso-8859-1"?


File Source for OWLVocabulary.php


# Source for file OWLVocabulary.php

Documentation is available at OWLVocabulary.php

```
1. <?PHP
2. /**
3. * ----------------------------------------------------------------------------------
4. * Class: OWLVocabulary
5. * ----------------------------------------------------------------------------------
6. *
7. * @package     ontModel
8. */

10. /**
11. * OWL vocabulary items
12. *
13. * <BR><BR>History:
14. * <LI>10-05-2004                : First version of this class.</LI>
15. *
16. * @version  V0.9.1
17. * @author Daniel Westphal <mail at d-westphal dot de>
18. *
19. *
20. * @package     ontModel
21. * @access    public
22. ***/
23. class OWLVocabulary extends OntVocabulary
24. {

26. /**
27. * Answer the resource that represents the class 'class' in this vocabulary.
28. *
29. * @return    object ResResource
30. * @access    public
31. */
32. function ONTCLASS()
33. {
34. return new ResResource(OWL_NS.'Class');
35. }

37. /**
38. * Answer the predicate that denotes the domain of a property.
39. *
40. * @return    object ResProperty
41. * @access    public
42. */
43. function DOMAIN()
44. {
45. return new ResProperty(RDF_SCHEMA_URI.RDFS_DOMAIN);
46. }


49. /**
50. * Answer the predicate that denotes comment annotation on an ontology element.
51. *
52. * @return    object ResProperty
53. * @access    public
54. */
55. function COMMENT()
56. {
57. return new ResProperty(RDF_SCHEMA_URI.RDFS_COMMENT);
58. }

60. /**
61. * Answer the predicate that denotes isDefinedBy annotation on an ontology element
62. *
63. * @return    object ResProperty
64. * @access    public
65. */
66. function IS_DEFINED_BY()
67. {
68. return new ResProperty(RDF_SCHEMA_URI.RDFS_IS_DEFINED_BY);
69. }

71. /**
72. * Answer the predicate that denotes label annotation on an ontology element
73. *
74. * @return    object ResProperty
75. * @access    public
76. */
77. function LABEL()
78. {
79. return new ResProperty(RDF_SCHEMA_URI.RDFS_LABEL);
80. }

82. /**
83. * Answer the predicate that denotes the domain of a property.
84. *
85. * @return    object ResProperty
86. * @access    public
87. */
88. function RANGE()
89. {
90. return new ResProperty(RDF_SCHEMA_URI.RDFS_RANGE);
91. }

93. /**
94. * Answer the predicate that denotes seeAlso annotation on an ontology element
95. *
96. * @return    object ResProperty
97. * @access    public
98. */
99. function SEE_ALSO()
100. {
101. return new ResProperty(RDF_SCHEMA_URI.RDFS_SEE_ALSO);
102. }

104. /**
105. * Answer the predicate that denotes that one class is a sub-class of another.
106. *
107. * @return    object ResProperty
108. * @access    public
109. */
110. function SUB_CLASS_OF()
111. {
112. return new ResProperty(RDF_SCHEMA_URI.RDFS_SUBCLASSOF);
113. }

115. /**
116. * Answer the predicate that denotes that one property is a sub-property of another.
117. *
118. * @return    object ResProperty
119. * @access    public
120. */
121. function SUB_PROPERTY_OF()
122. {
123. return new ResProperty(RDF_SCHEMA_URI.RDFS_SUBPROPERTYOF);
124. }


128. function ANNOTATION_PROPERTY()
129. {
130. return new ResProperty(OWL_NS . 'AnnotationProperty');
131. }

133. function ALL_DIFFERENT()
134. {
135. return new ResProperty(OWL_NS . 'AllDifferent');
136. }

138. function ALL_VALUES_FROM()
139. {
140. return new ResProperty(OWL_NS . 'allValuesFrom');
141. }

143. function BACKWARD_COMPATIBLE_WITH()
144. {
145. return new ResProperty(OWL_NS . 'backwardCompatibleWith');
146. }

148. function CARDINALITY()
149. {
150. return new ResProperty(OWL_NS . 'cardinality');
151. }

153. function COMPLEMENT_OF()
154. {
155. return new ResProperty(OWL_NS . 'complementOf');
156. }

158. function DATATYPE()
159. {
160. return new ResProperty(OWL_NS . 'Datatype');
161. }

163. function DATATYPE_PROPERTY()
164. {
165. return new ResProperty(OWL_NS . 'DatatypeProperty');
166. }

168. function DATA_RANGE()
169. {
170. return new ResProperty(OWL_NS . 'DataRange');
171. }

173. function DATATYPE_RESTRICTION()
174. {
175. return new ResProperty(OWL_NS . 'DatatypeRestriction');
176. }

178. function DEPRECATED_CLASS()
179. {
180. return new ResProperty(OWL_NS . 'DeprecatedClass');
181. }

183. function DEPRECATED_PROPERTY()
184. {
185. return new ResProperty(OWL_NS . 'DeprecatedProperty');
186. }

188. function DISTINCT_MEMBERS()
189. {
190. return new ResProperty(OWL_NS . 'distinctMembers');
191. }

193. function DIFFERENT_FROM()
194. {
195. return new ResProperty(OWL_NS . 'differentFrom');
196. }

198. function DISJOINT_WITH()
199. {
200. return new ResProperty(OWL_NS . 'disjointWith');
201. }

203. function EQUIVALENT_CLASS()
204. {
205. return new ResProperty(OWL_NS . 'equivalentClass');
206. }

208. function EQUIVALENT_PROPERTY()
209. {
210. return new ResProperty(OWL_NS . 'equivalentProperty');
211. }

213. function FUNCTIONAL_PROPERTY()
214. {
215. return new ResProperty(OWL_NS . 'FunctionalProperty');
216. }

218. function HAS_VALUE()
219. {
220. return new ResProperty(OWL_NS . 'hasValue');
221. }

223. function INCOMPATIBLE_WITH()
224. {
225. return new ResProperty(OWL_NS . 'incompatibleWith');
226. }

228. function IMPORTS()
229. {
230. return new ResProperty(OWL_NS . 'imports');
231. }

233. function INTERSECTION_OF()
234. {
235. return new ResProperty(OWL_NS . 'intersectionOf');
236. }

238. function INVERSE_FUNCTIONAL_PROPERTY()
239. {
240. return new ResProperty(OWL_NS . 'InverseFunctionalProperty');
241. }

243. function INVERSE_OF()
244. {
245. return new ResProperty(OWL_NS . 'inverseOf');
246. }

248. function MAX_CARDINALITY()
249. {
250. return new ResProperty(OWL_NS . 'maxCardinality');
251. }

253. function MIN_CARDINALITY()
254. {
255. return new ResProperty(OWL_NS . 'minCardinality');
256. }

258. function NOTHING()
259. {
260. return new ResProperty(OWL_NS . 'Nothing');
261. }

263. function OBJECT_CLASS()
264. {
265. return new ResProperty(OWL_NS . 'ObjectClass');
266. }

268. function OBJECT_PROPERTY()
269. {
270. return new ResProperty(OWL_NS . 'ObjectProperty');
271. }

273. function OBJECT_RESTRICTION()
274. {
275. return new ResProperty(OWL_NS . 'ObjectRestriction');
276. }

278. function ONE_OF()
279. {
280. return new ResProperty(OWL_NS . 'oneOf');
281. }

283. function ON_PROPERTY()
284. {
285. return new ResProperty(OWL_NS . 'onProperty');
286. }

288. function ONTOLOGY()
289. {
290. return new ResProperty(OWL_NS . 'Ontology');

292. }

294. function PRIOR_VERSION()
295. {
296. return new ResProperty(OWL_NS . 'priorVersion');
297. }

299. function PROPERTY()
300. {
301. return new ResProperty(OWL_NS . 'Property');
302. }

304. function RESTRICTION()
305. {
306. return new ResProperty(OWL_NS . 'Restriction');
307. }

309. function SAME_AS()
310. {
311. return new ResProperty(OWL_NS . 'sameAs');
312. }

314. function SAME_CLASS_AS()
315. {
316. return new ResProperty(OWL_NS . 'sameClassAs');
317. }

319. function SAME_INDIVIDUAL_AS()
320. {
321. return new ResProperty(OWL_NS . 'sameIndividualAs');
322. }

324. function SAME_PROPERTY_AS()
325. {
326. return new ResProperty(OWL_NS . 'samePropertyAs');
327. }

329. function SOME_VALUES_FROM()
330. {
331. return new ResProperty(OWL_NS . 'someValuesFrom');
332. }

334. function SYMMETRIC_PROPERTY()
335. {
336. return new ResProperty(OWL_NS . 'SymmetricProperty');
337. }

339. function THING()
340. {
341. return new ResProperty(OWL_NS . 'Thing');
342. }

344. function TRANSITIVE_PROPERTY()
345. {
346. return new ResProperty(OWL_NS . 'TransitiveProperty');
347. }

349. function UNION_OF()
350. {
351. return new ResProperty(OWL_NS . 'unionOf');
352. }

354. function VERSION_INFO()
355. {
356. return new ResProperty(OWL_NS . 'versionInfo');
357. }

359. function NAMESPACE()
360. {
361. return OWL_NS;
362. }

364. /**
365. * Answer the predicate that denotes the rdf:type property.
366. *
367. * @return    object ResProperty
368. * @access    public
369. */
370. function TYPE()
371. {
372. return new ResProperty(RDF_NAMESPACE_URI.RDF_TYPE);
373. }
374. }
375. ?>
```

Documentation generated on Thu, 7 Jul 2005 13:42:36 +0200 by phpDocumentor 1.3.0RC3
